# Forensic parameters of 41 Y-STR loci in Shandong Han individuals and comparison with 42 other populations

**DOI:** 10.1080/20961790.2021.1963397

**Published:** 2021-10-04

**Authors:** Anqi Chen, Li Luo, Ruiyang Tao, Suhua Zhang, Chengtao Li

**Affiliations:** a Department of Forensic Medicine, School of Basic Medical Sciences, Shanghai Medical College, Fudan University, Shanghai, China; b Shanghai Key Laboratory of Forensic Medicine, Shanghai Forensic Service Platform, Academy of Forensic Science, Ministry of Justice, Shanghai, China

The Y-chromosomal short tandem repeat polymorphisms (Y-STRs) are the male-specific markers. The characteristics of paternal lineages make it a valuable tool for tracing familial relationships [1–3]. Y-STR analysis has been widely used for identifying genealogical DNA testing and to identify missing persons, assess paternal relationships, and investigate sexual assault cases [4, 5]. China is a united and multinational country with vast territory. Populations of different geographical and ethnic groups might carry different genetic backgrounds; therefore, it is meaningful to learn the genetic backgrounds and inter-population relationships of China. The Shandong Province encompasses the eastern coastal areas and the lower reaches of the Yellow River. The inland borders the Hebei, Henan, Anhui, and Jiangsu provinces from north to south. In addition, since it is one of the most deve­loped provinces, population migration might also influence the genetic background of the area.

Analysis of allele frequencies of Y-STRs is indispensable for forensic reference database construction and population genetics. To determine the genetic polymorphism and haplotype frequency of 41 Y-STRs in the Shandong Han population in China, 567 unrelated male individuals were recruited for the present study. Genomic DNA was extracted from the bloodstain samples, using the Chelex-100 protocol described by Walsh et al. [6], and quantified using a Qubit fluorometer (Life Technologies, Carlsbad, CA, USA). Extracted DNA was stored at −80 °C until use. All samples were collected with the approval of the Ethics Committee of Academy of Forensic Science, Ministry of Justice, China (approval code: No. SJY2019-W005). Informed consent was obtained from all individual participants included in this study.

The Goldeneye® Y-Plus Kit (Peoplespot, Beijing, China) was used to genotype Y-STRs, including 41 Y-STR loci (DYS456, DYS549, DYS439, DYS19, DYS392, DYS643, DYS447, DYS557, DYS391, DYS388, DYS570, DYS635, DYS448, DYS437, DYS527, DYS444, DYS393, DYS389I, DYS390, DYS389II, DYS438, DYS576, DYS645, DYF404S1, DYS460, DYS458, DYS481, DYS385, DYS449, DYS596, Y-GATA-H4, DYS533, DYS627, DYS518, DYF387S1, DYS593 and DYS522). Fluorescent multiplex polymerase chain reaction (PCR) was conducted according to the manufacturer’s protocols. The STRs were genotyped using a 3500 ABI Prism Genetic Analyzer (Applied Biosystems, Foster City, CA, USA), and the resulting data were analyzed using GeneMapper ID-X software (Applied Biosystems).

Haplotype and allele frequencies were calculated using the direct counting method. Forensic parameters were calculated using the formulas described by Nei [7]. Haplotype match probability (HMP) was calculated using the formula,$\;\mathrm{HMP}=\sum_{i=1}^{k}{\mathrm{pi}}^{2}$, where pi refers to the frequency of a haplotype and *k* refers to the number of haplotypes. Haplotype diversity (HD) or genetic diversity (GD) was calculated using the formula, $HD(GD)=\frac{n}{n-1}\times (1-\sum_{i=1}^{k}{\mathrm{pi}}^{2})$, where *n* refers to the sample size. Discrimination capacity (DC) was calculated using the formula, $=\frac{k}{\sum_{i=1}^{k}\left(pi\times n\right)}$. Population pairwise genetic distances (Rst) and multidimensional scaling (MDS) analyses were performed using the online statistical tool available on the YHRD website (https://yhrd.org/amova). Phylogenetic analyses were conducted on MEGA 7.0 software [8] using the unweighted pair group method with arithmetic mean (UPGMA) method [9].

As shown in Supplementary Tables S1 and S2, 563 unique haplotypes were detected. Only one non-unique haplotype was detected in two individuals, and the haplotype frequencies ranged from 0.1864% (1/567) to 0.3527% (2/567). The allele frequencies are shown in Supplementary Table S3; DYS385 was the most polymorphic STR (GD = 0.9618), followed by DYF387S1 (GD = 0.9425), DYS527 (GD = 0.9378), and DYF404S1 (GD = 0.8975). It has been reported that the discrimination capacity and haplotype diversity can be increased if more markers are included [1, 4, 10]. Forensic parameters were calculated for seven different haplotype sets to determine the resolution of the new Y-STR markers. As expected, the number of unique haplotypes increased with the increasing number of loci. Only 327 unique haplotypes were detected using the minimal haplotype set (9 Y-STR loci), and 543 unique haplotypes were identified using the 29 Y-STR loci set. The number of unique haplotypes further increased to 563 after adding 12 additional markers, and the most frequent haplotypes were shared by only four individuals. The DC values was only 0.5767 for the minimal haplotype set, and it dramatically increased to 0.9965 for the Goldeneye Y-Plus kit (41 Y-STR loci) (Supplementary Table S2). The results suggested that a large value was produced by a larger panel size, and that the 41 Y-STRs could enable further efforts to improve the discrimination capability.

To compare the Rst values of the Shandong Han population and others, 42 population data points were extracted from the YHRD website (https://yhrd.org/amova), which included 27 Chinese ethnic groups and 15 populations of different nationalities. The results showed significant differences between the Shandong Han population and others (Supplementary Tables S4 and S5). The largest genetic distance to the Shandong Han population was exhibited by the Aba Tibetan population (Rst = 0.3229, *P* < 0.0001), followed by the Garzê Tibetan (Rst = 0.3074, *P* < 0.0001), Chamdo Tibetan (Rst = 0.2824, *P* < 0.0001), and Sichuan Tibetan populations (Rst = 0.2478, *P* < 0.0001). The closest genetic distance was exhibited by the Shaanxi Han (Rst = 0.0045, *P* = 0.0028), Jiangsu Han (Rst = 0.0048, *P* = 0.0019), and Jiangxi Han populations (Rst = 0.0100, *P* < 0.0001) (Supplementary Table S4). The MDS plots showed that the Shandong Han population was clustered with the populations of East China (Shanghai Han, Shaanxi Han, Jiangsu Han, etc.), and was away from the populations of the Middle-South China (Guangdong Han), South-west China (Xinjiang and Qinghai), and North-west China (Tibetan, Guiyang Yi and Guizhou Bouyei). Furthermore, all 11 populations of Tibetan highlanders clustered in one dimension (Supplementary Figure S1A). These results suggested that the geographic location played an important role in genetic distance, and the regions of geographic proximity shared more genetic similarities. Both the Hunan Miao and Hunan Tujia populations resided in Hunan Province, and the evolutionary relationship was close (Supplementary Figure S1B). The same result was also reflected by the MDS plot, wherein the data points of the Hunan Miao population overlapped that of the Hunan Tujia population (Supplementary Figure S1A). Comparable results were also obtained at the global scale; the lowest genetic distance value was exhibited by the Swiss and Belgian populations (Rst = −0.0007, *P* = 0.5524), and the highest genetic distance value was exhibited by the Irish and African American populations (Rst = 0.3438, *P* < 0.0001) (Supplementary Table S5, Figure S2A and B). Interesting, although the Guizhou Province is located in the Southwest, the Miao population was genetically closer to the other Chinese Han populations. The same observation was also reported by Tao et al. [11] and Zhang et al. [10], who attributed the findings to the intermarriage between the populations.

Owing to global migration and labour market construction, many people have moved to prosperous areas. Migration is likely to result in a demographic transition [12, 13]. As shown in Supplementary Figure S2A, the distribution of the Americans alone was dispersed. The European American population clustered with the populations from the European countries, and the Asian American population grouped with populations from Asia. The phylogenetic tree yielded similar results (Supplementary Figure S2B). Although China is a multi-ethnic country, similar population characteristics could also be observed among Chinese ethnic groups. Most of the Han populations gathered in one dimension, and all Tibetan populations converged in the upper right dimension (Supplementary Figure S1A). As for the other ethnic groups, the populations of different ethnic minorities seldom clustered together (Supplementary Figure S1B).

In conclusion, the present study yielded valuable genetic data on 41 Y-STRs in the Chinese Shandong Han population. The forensic parameters demonstrated that the Y-STR marker sets were highly polymorphic, and hence constituted a promising panel for familial relationship estimation in the fields of forensics and population geography. The Shandong Han population was most similar to the Shaanxi Han population, and it was quite different from geographically distant populations such as the Irish population, and the other ethnic minorities such as the Tibetan population. The results of genetic distance suggested that the population differed according to the geographic location and human ancestry.

## Supplementary Material

Supplemental Material
