# Supplementary figures and images for: Forensic parameters of 41 Y-STR loci in Shandong Han individuals and comparison with 42 other populations
Source: Forensic Sci Res. 2021 Oct 4;7(4):823–5. doi: 10.1080/20961790.2021.1963397 (PMC9930784; doi:10.1080/20961790.2021.1963397)

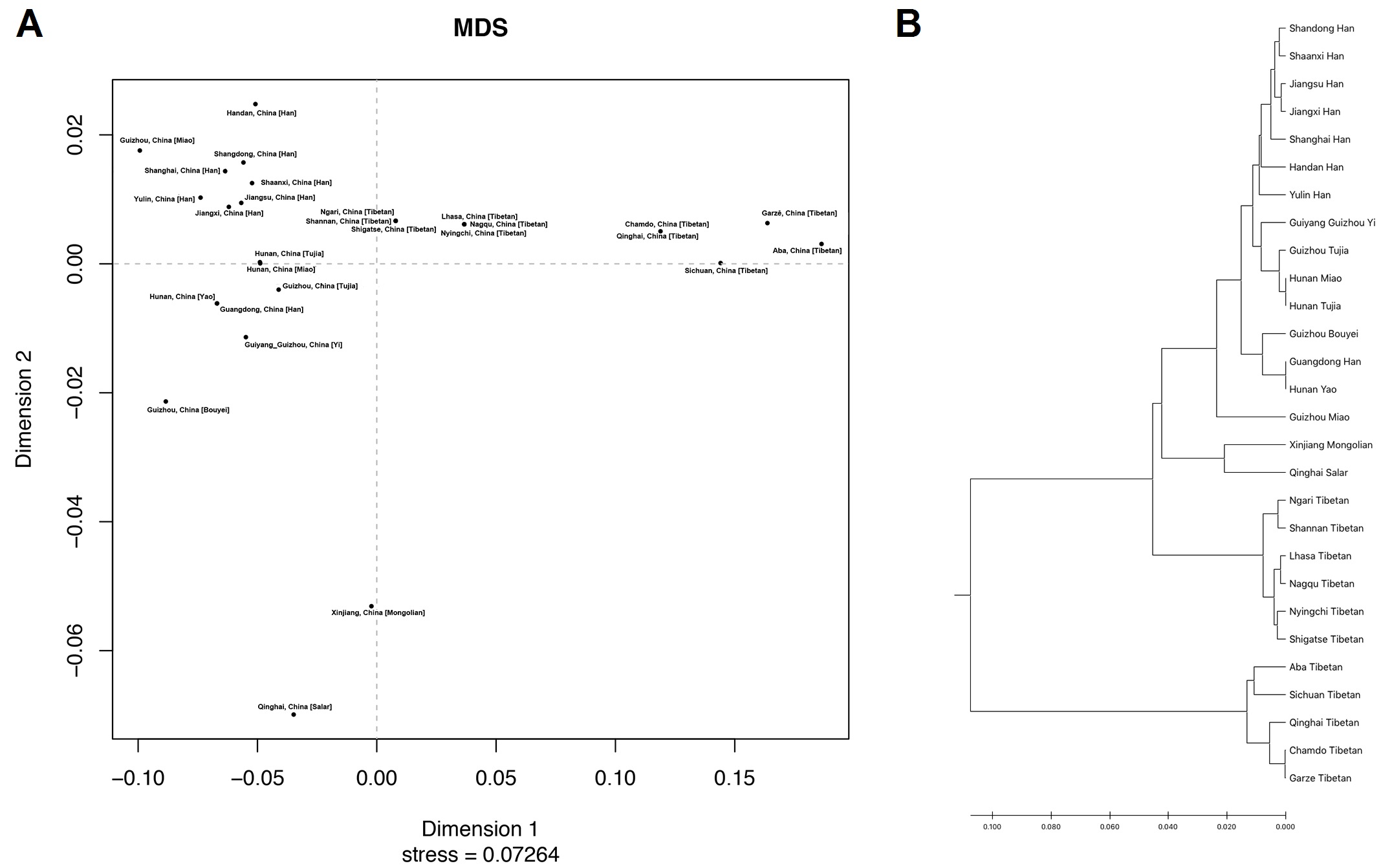

Supplement: Supplemental Material [file TFSR_A_1963397_SM6896.zip › Figure_S1.jpg]

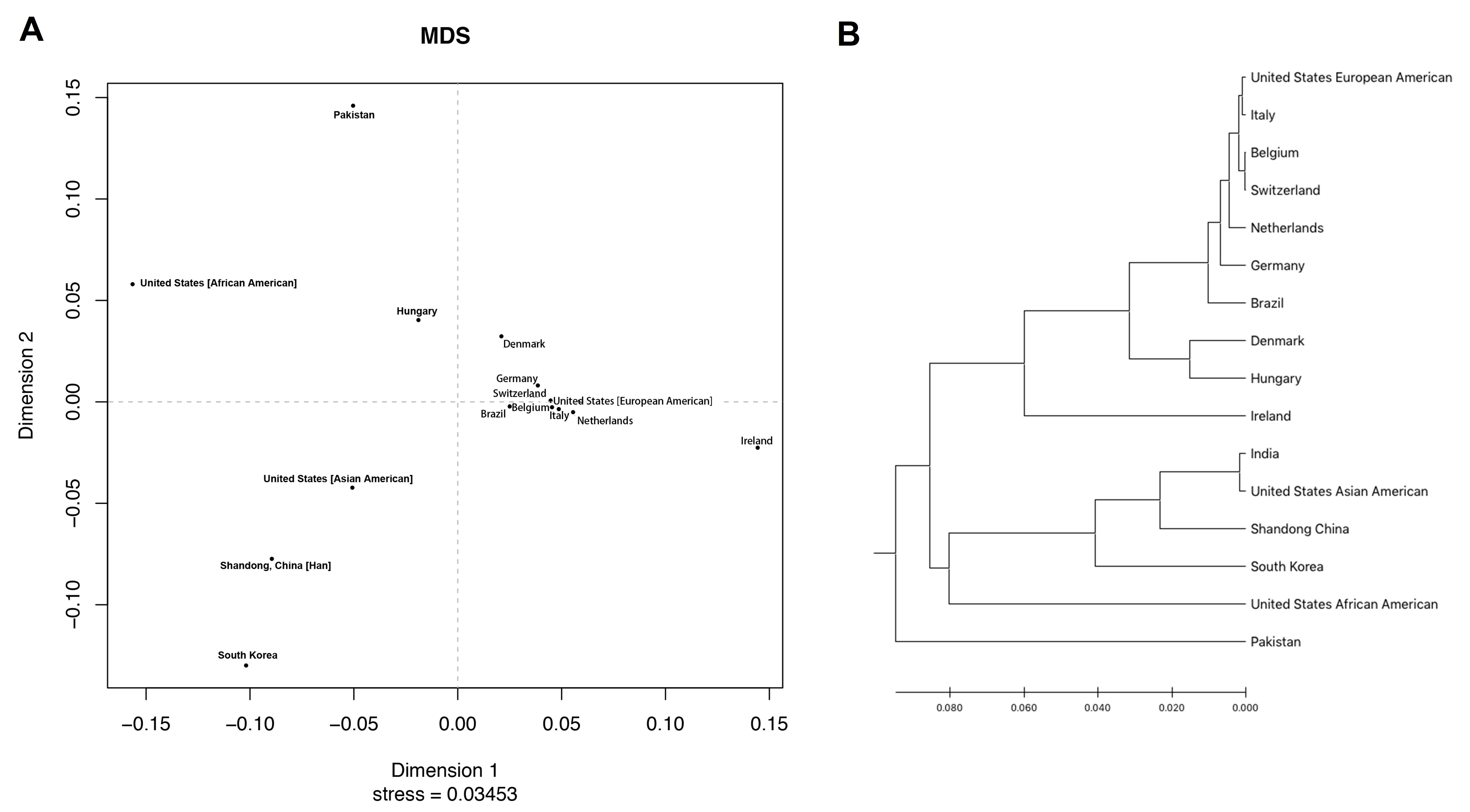

Supplement: Supplemental Material [file TFSR_A_1963397_SM6896.zip › Figure_S2.jpg]
